# Avascular necrosis in pediatric rheumatic diseases: an Italian retrospective multicentre study

**DOI:** 10.1186/s13052-025-01845-8

**Published:** 2025-01-29

**Authors:** Ivan Taietti, Federico Zini, Emilio Amleto Conti, Enrica Cristini, Irene Borzani, Giulia Ramponi, Claudia Bracaglia, Raffaele Pecoraro, Riccardo Papa, Jessica Tibaldi, Serena Pastore, Gabriele Simonini, Marco Cattalini, Antonella Meini, Achille Marino, Stefano Lanni, Francesca Minoia, Giovanni Filocamo

**Affiliations:** 1 Pediatric Immuno-Rheumatology Unit, Fondazione IRCCS Ca’ Granda Ospedale Maggiore Policlinico, Milan, Italy; 2 University of Pavia, Pavia, Italy; 3 University of Milan, Milan, Italy; 4 Department of Orthopaedics and Traumatology, Fondazione IRCCS Ca’ Granda Ospedale Maggiore Policlinico, Milan, Italy; 5 Pediatric Radiology Unit, Fondazione IRCCS Ca’ Granda Ospedale Maggiore Policlinico, Milan, Italy; 6 Pediatric Unit, Fondazione IRCCS San Gerardo, Monza, IT Italy; 7 Division of Rheumatology, IRCCS Ospedale Pediatrico Bambino Gesù, Rome, Italy; 8 Pediatric Clinic and Rheumatology, IRCCS Istituto Giannina Gaslini, University of Genoa, Genoa, Italy; 9 Institute for Maternal and Child Health - IRCCS “Burlo Garofolo”, Trieste, Italy; 10 Rheumatology Unit, ERN-ReCONNET center Meyer Children’s Hospital IRCCS, University of Florence, Florence, Italy; 11 Pediatric Clinic and Molecular Medicine Institute ‘A. Nocivelli’, University of Brixia, Brescia, Italy; 12 Unit of Pediatric Rheumatology, ASST G. Pini-CTO, Milan, 20122 Italy

**Keywords:** Avascular necrosis (AVN), Systemic glucocorticoids, Pediatric rheumatology, Pediatric rheumatic diseases

## Abstract

**Background:**

Atraumatic avascular necrosis (AVN) is a severe condition that may complicate the course of rheumatic diseases and contribute to long-term damage. However, there is a lack of evidence on this rare event in pediatric rheumatology. The aim of our study was to evaluate the occurrence of avascular necrosis in the context of rheumatologic diseases in Italy and to describe the main demographic and clinical features of AVN patients, with a particular focus on treatment background.

**Methods:**

All centres part of the Italian Society of Pediatric Rheumatology were invited to participate in a retrospective case collection of children with rheumatic diseases complicated by a pediatric-onset AVN. Demographic, clinical, laboratory and imaging data were recorded, together with outcome and treatment background, particularly steroid exposure. Population collected was further evaluated according to the different underlying rheumatologic disease and to the time of AVN onset.

**Results:**

Fourteen patients (SLE = 7; JIA = 4; others = 3) were collected from 7 centres. Females were predominantly affected (71%) with a median age at AVN diagnosis of 14.3 years. Multifocal involvement was mostly reported (93%), mainly involving femoral heads (44%) and knees (28%). All patients had a severe rheumatologic background and received systemic glucocorticoids with a median cumulative prednisone equivalent dose of 457.5 mg/kg. In all patients but one imaging showed persistence of abnormalities, despite the complete resolution of symptoms in 6 of them. Bisphosphonates were the most used therapeutic approach; orthopedic surgery was required in 2 cases.

**Conclusions:**

Despite its rarity, AVN may be a severe complication of pediatric rheumatic diseases. Active monitoring is crucial to promptly identify patients and to prevent long-term damage. Prospective large sample studies are required to better understand the impact of steroid exposure and its complex interplay with other potential contributing factors.

## Introduction

Avascular necrosis (AVN) is a massive necrosis of the bone and red medulla caused by either traumatic events or atraumatic risk factors, mainly glucocorticoid use [1]. Atraumatic AVN is influenced by several genetic and metabolic factors together with physical blood flow restrictive events [2] and mostly affects long-bone epiphyses, in particular femoral head and condyles, distal end of the tibia, and humeral head. AVN often shows a multifocal distribution [3]. Once the condition progresses to advanced stages and destruction occurs, surgical interventions, including bone grafting or hip replacement could be required [4].

AVN has been also reported in rheumatologic disorders, especially in systemic lupus erythematosus (SLE), as a consequence of insufficient bone perfusion [5]. The reported prevalence of AVN in patients with SLE ranges between 10 and 44% [6]. Glucocorticoids (GCs) use was recognized as the principal risk factor for AVN [7] in SLE, probably due to its known effect on endothelial cells, hypercoagulability, fat cell hypertrophy, and the inhibition of angiogenesis through the reduction of blood flow and oxygen delivery in micro-vessels [5]. Although some cases of AVN have been associated to intra-articular steroid injection (IACI) [1], systemic GCs are largely the most reported factor associated with the development of AVN, and risk seems to correlate both with the duration of GC treatment and with the amount of GCs received [8, 9]. However, so far attempts to identify a safe level of average or maximum daily dose of GCs, as well as a maximum duration of steroidal treatment failed, mainly due to the multitude of confounding variables in the studies performed [1, 10, 11].

Reports on AVN in children with rheumatic diseases are limited to single case reports and small case series, with a lack of evidence on this rare event in pediatric rheumatology [12]. Against this background, our study is aimed to investigate the occurrence of AVN in children with underlying rheumatic disease in Italy and to describe the demographic and clinical characteristics of AVN patients with a particular focus on treatment background.

## Methods

### Study population

All the centers part of the Italian Society of Pediatric Rheumatology were invited to participate to the study and a preliminary survey was made to identify those centers that observed cases of AVN in children with underlying rheumatic diseases from 2000 to date. To be included patients should have received a diagnosis of AVN, confirmed by magnetic resonance imaging (MRI) before the age of 18, and should be affected by a rheumatic disease. Patients with previous orthopedic surgery in the site of AVN and/or previous diagnoses of congenital hip dysplasia, Gaucher disease, hemoglobinopathies, malignant carcinoma, and/or previous hematopoietic or solid transplantation were excluded.

The centers who accepted to participate and observed at least one case of AVN were asked to complete a standardized case report form (CRF), which collected data on demographical features, characteristics of AVN and the rheumatologic background of the underlying disease.

In particular, data regarding AVN included date of diagnosis, number and type of sites involved, imaging performed, specific treatment used and outcome. Information collected on the rheumatologic background were the date of onset, major clinical manifestations, a physician global evaluation of disease activity (DA) at onset, during the course of the disease and at the AVN onset (low-moderate-high), the presence of flares, any complication related to the underlying disease or the treatment received, any intensive care unit (ICU) admission. Among laboratory features, antinuclear antibodies (ANA), anti-double strained DNA (dsDNA) antibodies, C3 and C4 fractions, antiphospholipid antibodies (lupus anticoagulant, antiβ2glicoprotein1, anticardiolipin), Extractable Nuclear Antigen Antibodies (ENA), antineutrophil cytoplasmic antibodies (ANCA), and Coomb’s test were recorded. Treatment background, especially the GC use, was carefully investigated. GC therapy was converted in cumulative dose of prednisone equivalent mg/kg to allow comparisons among patients. We referred to the Meikle table for GC conversion [13]. Similarly to Felson et al. [14], we analyzed the GC cumulative dosage at 1, 3, 6 and 12 months after steroidal beginning and the administration or not of GC pulses.

The present study was conducted following the principles of the Declaration of Helsinki and the study protocol was approved by the local Institutional Review Board.

### Statistical analyses

Demographic and clinical characteristics were reported as median and interquartile range (IQR) for continuous variables and percentages for categorical variables, as appropriate. Differences in continuous variables between groups were assessed by performing Mann-Whitney non-parametric test, while association between categorical variables was tested by using chi-square or Fisher’s exact tests as appropriate.

AVN features were compared among patients with different rheumatologic backgrounds and in particular between the two most common conditions, i.e. systemic lupus erythematosus (SLE) and systemic juvenile idiopathic arthritis (sJIA). Furthermore, patients with “early-onset AVN” (defined as an AVN onset within one year from rheumatologic diagnosis) were compared to the rest of the population in order to evaluate potential contributors to a more aggressive form.

## Results

Fourteen patients were collected from seven Italian centers. Females were predominantly affected (71%), with a M: F ratio of 2:5. The ethnicity most represented was Caucasian (71%), followed by Asiatic, Hispanic, and Black people. Globally, the median age at the onset of the underlying rheumatologic disease was 12.2 years (IQR: 7.9–13.4) [Table 1].

**Table 1:** Demographic and main rheumatologic background of collected patients with AVN

	Patients with AVN (*N* 14)
**Female**	10 (71%)
**Median (IQR) age at AVN diagnosis**, years	14.3 (13.2–15.4)
**Median (IQR) age at onset of the underlying disease**, years	12.2 (7.9–13.4)
**Median disease duration at AVN diagnosis**, years	1.8 (0.7–4.5)
**Rheumatologic background**	
- SLE - JIA - APS - PAN - SAPHO syndrome - UCTD	7 (50%) 4 (29%) 1 (7%) 1 (7%) 1 (7%) 1 (7%)
**Disease activity at onset of the underlying disease**	
Mild Moderate Severe	0 (0%) 4 (29%) 10 (71%)
**Disease activity at AVN diagnosis**	
Mild Moderate Severe	6 (43%) 7 (50%) 1 (7%)
**Autoantibodies profile**	
- ANA - anti-dsDNA - LAC - anti-beta2GP1 - anti-cardiolipin - anti-Smith - anti-Ro/SSA - anti-La/SSB - p-ANCA - hypocomplementemia - direct Coomb’s test positivity	8 (57%) 7 (50%) 2 (14%) 2 (14%) 2 (14%) 2 (14%) 2 (14%) 1 (7%) 1 (7%) 7 (50%) 1 (7%)

Data are expressed as number (percentage) unless otherwise specified. *ANA*, anti-nuclear anti-bodies. *Anti-beta2GP1*, anti-beta2-glicoprotein 1 antibodies. *Anti-dsDNA*, anti-double strained DNA. *APS*, anti-phospholipid syndrome. *IQR*, interquartile range. *sJIA*, systemic juvenile idiopathic arthritis. *LAC*, lupus anti-coagulant. *PAN*, polyarteritis nodosa. *SAPHO*, synovitis, acne, pustulosis, hyperostosis, and osteitis. *SLE*, systemic lupus erythematosus. *UCTD*, undifferentiated connective tissue disease

The median age at the diagnosis of AVN was 14.3 years (IQR 13.2–15.4) with a disease median duration of 1.8 years (IQR 0.7–4.5). As reported in Table 2, AVN was found in 39 different sites, and was multiple in almost all patients (93%): most patients (7) had 2 sites involved, while 3 patients were affected in 3 sites and 2 in at least 4 sites. The sites most commonly affected were femoral head and knee. Of note, AVN involvement was symmetric in 71% of patients. Patients were mainly symptomatic (71%) and pain was the most reported symptom (100%), followed by functional limitation (70%) and joint swelling (40%).

**Table 2:** Clinical characteristics of AVN

	Patients with AVN (*N* 14)
**Multiple site involvement**	13 (93%)
**Symmetric involvement**	10 (71%)
**Localization**	
- femoral head - knee - ankle - shoulder - elbow	17 (44%) 11 (28%) 8 (20%) 2 (5%) 1 (3%)
**Clinical manifestations**	
- Asymptomatic - Pain - Swelling - Limitation of motion	4 (29%) 10 (71%) 4 (29%) 7 (50%)

Data are expressed as number (percentage) unless otherwise specified. *AVN*, avascular necrosis

In our population, the rheumatologic condition mostly represented was SLE in 7 patients, followed by sJIA (4 patients), and anti-phospholipid syndrome (APS), polyarteritis nodosa (PAN), Synovitis, Acne, Pustulosis, Hyperostosis, Osteosis (SAPHO) syndrome and undifferentiated connective tissue disease (UCTD) with one patient each. The rheumatologic course was complicated by macrophage activation syndrome (MAS) and by thrombotic microangiopathy in 4 and 1 case, respectively; four patients required ICU admission. The DA of the underlying disease was reported as moderate or severe at onset in all patients, and during the global course of the disease in most of them (79%). Interestingly, 43% of patients developed AVN once in remission. Patients with a moderate-severe DA during the course of the underlying disease presented AVN earlier compared to patients with mild DA, although difference did not reach statistical significance (569 days [IQR:254–1327] vs. 1454 days [IQR: 300–2311]; *p*-value: 0.49).

All patients received systemic GCs, and 13 of them were treated with methylprednisolone (MPN) pulses (3 to 6 pulses at 30 mg/kg/day, maximum 1 gr/day). Multiple IACI were reported in 2 sJIA patients: of note, both patients had a severe onset and course of the rheumatologic disease and required high-doses of systemic MPN. Table 3 reports the details on the use of GCs in our cohort. At AVN diagnosis, the median cumulative GC dose was 457.5 mg/kg (IQR 259.5–849), and 71% of patients were still receiving GCs with a median dose of 288 mg/kg (IQR 158–451.7). Of note, almost all patients (93%) developed clinical signs of steroidal toxicity. The non-steroidal therapeutic background and the ongoing treatment at AVN diagnosis is summarized in Table 4. The median number of medications reported as associated to GCs was 4 (IQR 3–6) for each patient, with biological agents used in 6 cases (43%), mainly sJIA.

**Table 3:** Details on glucocorticoid background in patients with AVN

	Patients with AVN (*N* 14)
**MPN pulses** (30 mg/kg/day, maximum 1 gr)	13 (93%)
**Cumulative GC dose** (mg/kg [median (IQR)])	
At 1 month from disease onset: At 3 months from disease onset: At 6 months from disease onset: At 12 months from disease onset: At AVN diagnosis:	117 (103–129) 171 (138–225) 209 (164–270) 283 (239–429) 455 (245–838)
**Patients receiving GC at AVN diagnosis**	10 (71%)
**Median daily GC dose at AVN diagnosis** (mg/kg [median (IQR)])	457.5 (259.5–849)
**Clinical signs of GC toxicity**	13 (93%)
- Cushing habitus - Failure to thrive - Hypertension - Diabetes mellitus - Osteopenia	12 (92%) 2 (15%) 6 (46%) 1 (8%) 7 (54%)

Data are expressed as number (percentage) unless otherwise specified. AVN, avascular necrosis; GC, glucocorticoid; MPN, methylprednisolone

**Table 4:** Details on non-steroidal background in patients with AVN

	Patients with AVN (*N* 14)	
	Non-steroidal treatment received before AVN diagnosis	Non-steroidal treatment ongoing at AVN diagnosis
**Conventional DMARDs** - mofetil mycophenolate - Hydroxychloroquine - Azathioprine - Cyclophosphamide - Methotrexate - Cyclosporine A - IVIG	13 (93%) 7 (50%) 7 (50%) 6 (43%) 6 (43%) 6 (43%) 5 (36%) 3 (21%)	10 (71%) 3 (21%) 6 (43%) 2 (14%) 1 (7%) 4 (29%) 1 (7%) 0
**Biological DMARDs** - anakinra - canakinumab - adalimumab - etanercept - tocilizumab - rituximab - secukinumab	6 (43%) 4 (29%) 2 (14%) 3 (21%) 4 (29%) 4 (29%) 1 (7%) 1 (7%)	3 (21%) 0 0 2 (14%) 0 1 (7%) 0 0

Data are expressed as number (percentage) unless otherwise specified. *AVN*, avascular necrosis; *DMARD*, disease-modifying antirheumatic drug

Therapeutic approaches for AVN and relative outcomes are detailed in Table 5. In all patients but one imaging showed a persistence of abnormalities, despite a complete resolution of symptoms in six of them. Six patients still complained of pain and functional limitations at last visit, and only one patient achieved both clinical and radiological remission. Bisphosphonate, hyperbaric oxygen and magnetic therapy were used in 4, 3 and 1 case, respectively. Two patients received orthopedic surgery. Interestingly, 7 patients (4 asymptomatic) did not receive any treatment. Globally a complete resolution of symptoms together with a normalization of imaging was reached in 5 patients (36%), while permanent sequelae were reported in 6 cases (43%). Considering the untreated patients, all of them resolved clinical symptoms, but only one normalized imaging findings. (Table 5).

**Table 5:** Specific treatments administered for AVN and outcome

Patient ID	Underlying disease	Age at disease onset	Age at AVN onset	Site of AVN	Total cumulative dosage of GC at AVN onset	Specific treatment for AVN	Outcome
1 (F)	SLE	12,2	12,9	Bilateral distal femoral epiphysis and metaphysis	231 mg/kg	Hyperbaric oxygen	Symptoms (pain, functional limitation) and abnormal imaging persistence
2 (F)	SLE	14,2	15,2	Bilateral femoral head	363 mg/kg	Hyperbaric oxygen and bisphosphonate	Symptoms (pain, functional limitation) and abnormal imaging persistence
3 (M)	sJIA	15,2	15,6	Left ulnar olecranon, bilateral humeral head, bilateral femoral head and bilateral proximal and distal tibial metaphysis	130 mg/kg	Hyperbaric oxygen and bisphosphonate	Symptoms relief
4 (F)	UCTD	8	15,7	Right ankle	2220 mg/kg	Orthopedic surgery	Symptoms (pain, functional limitation) and abnormal imaging persistence
5 (F)	SLE, APS	13,3	14,9	Bilateral distal femoral metaphysis	865 mg/kg	/	Abnormal imaging persistence
6 (F)	PAN	13,1	13,4	Bilateral tibial diaphysis, and proximal metaphysis and epiphysis. Bilateral femoral diaphysis and distal metaphysis	288 mg/kg	/	Symptoms relief and abnormal imaging persistence
7 (F)	sJIA	7,7	10,3	Right femoral head and bilateral talus	NA	Bisphosphonate	Symptoms (pain, functional limitation) and abnormal imaging persistence
8 (M)	SAPHO	13,6	14,4	Bilateral distal femoral metaphysis	158 mg/kg	/	Imaging normalization
9 (F)	SLE	11,3	17,5	Bilateral femoral head	497 mg/kg	/	Symptoms relief and abnormal imaging persistence
10 (F)	SLE	13,4	14	Bilateral femoral head	132 mg/kg	/	Symptoms relief and abnormal imaging persistence
11 (F)	SLE	12,5	14,3	Bilateral femoral head.	452 mg/kg	/	Abnormal imaging persistence
12 (M)	sJIA	3,4	8,7	Right talus. Bilateral femoral head.	1077 mg/kg	Magnetic therapy	Symptoms (pain) and abnormal imaging persistence
13 (M)	sJIA	2,5	6,1	Bilateral femoral head.	458 mg/kg	/	Abnormal imaging persistence
14 (F)	SLE	10	13,6	Bilateral femoral head.	758 mg/kg	Bisphosphonate and orthopedic surgery	Pain and swelling relief with functional limitation and abnormal imaging persistence

*APS*, anti-phospholipid syndrome. *AVN*, avascular necrosis *sJIA*, systemic juvenile idiopathic arthritis. *PAN*, polyarteritis nodosa. *SAPHO*, synovitis, acne, pustulosis, hyperostosis, and osteitis. *SLE*, systemic lupus erythematosus. *UCTD*, undifferentiated connective tissue disease

AVN was diagnosed earlier in SLE compared to sJIA, with a median duration of the rheumatologic disease at AVN onset of 1.6 years (IQR 0.7–3.6) and 3.1 years (IQR 0.9–4.9) respectively, although statistical significance was not reached (*p*-value: 0.65). All patients with SLE and sJIA received GC pulses, without any difference in the cumulative steroid dosage nor in the global DA between the two conditions. In 6 patients (43%) AVN was diagnosed within a year from the onset of the rheumatologic disease (*early-onset* AVN), whereas in the remaining group AVN occurred after a median of 3.6 years (IQR 2.6–6.2) The early-onset group included 3 SLE, 1 sJIA, 1 SAPHO and 1 PAN. We did not find any significant differences in the number of patients that received GC pulses. However, median total cumulative dosage of GC at AVN onset was significantly lower in the *early-onset* group (195 mg/kg vs. 812 mg/kg; *p*-value: < 0.05). Moreover, a greater number of patients with early-onset AVN were receiving both GCs (6 vs. 4; *p*-value: 0.040) and at least one disease-modifying antirheumatic drug (DMARD) (6 vs. 4; *p*-value: 0.040) at the time of AVN diagnosis compared to the rest of population.

## Discussion

Despite rare, AVN might be a severe complication of pediatric rheumatologic diseases. Given the high risk of long-term damage, improving knowledge about the role of potential contributors, especially steroid treatment, and increasing awareness on timely diagnosis and therapeutic approaches is crucial. However, so far, the evidence on AVN in pediatric rheumatology is still poor. AVN has been described more frequently in SLE patients [15–17]. Indeed, SLE was the most reported rheumatologic disorder in our population and the main features observed in our SLE patients were comparable to previously described cohorts [15], in which AVN was described as symmetrical, often insidious and asymptomatic in a relevant percentage of cases and mainly involving the femoral head.

Risk factors of AVN in the context of rheumatic diseases are difficult to define, and may include medications received and especially the steroidal load, features related to the underlying inflammatory condition, genetic predisposition and many other factors like peculiar anatomical features of the site involved, coagulation defects, endothelial cell damage, lipid abnormalities [1].

High-dose GCs are considered the main risk factor for AVN in patients with rheumatic diseases [15, 18]. Almost all patients in our cohort showed clinical signs of steroid toxicity, reflecting the significant dose of GCs received and the steroid sensitivity of this category of patients. In the study by Kallas et al. [19] 40 mg daily prednisone for 1 month was the strongest predictive factor for AVN, while MPN pulses did not increase AVN risk, suggesting cumulative steroidal dosage as the most relevant parameter related to GC use. Tsai et al. found that a cumulative GC dose up to 5 g was independently associated with the development of AVN in patients with autoimmune diseases, while hydroxychloroquine use for more than 0.6 years conferred a significant protection against its development, probably due to its steroid-sparing effect [9]. Unfortunately, only limited and non-conclusive data are so far available for pediatric population, mostly limited to children and adolescents with SLE. Yang et al. [15] observed that although both maximum daily prednisone dose and cumulative prednisone dose were significant in determining risk for AVN, the maximum daily prednisone was the most important risk factor for AVN in pediatric SLE. On the other hand, they did not find any differences in hydroxychloroquine use. IACI have been considered a risk factor for AVN, although the exact mechanism remains unclear [20]. In our cohort, only two patients with sJIA received IACI in joint sites of subsequent AVN, however both patients were also treated with high dose of systemic GCs.

Evidence regarding risk factors other than GCs is less robust. Data regarding the role of SLE are controversial. Although active SLE was identified in the past as independently associated to AVN [21], recent studies have supported the primary role of GC exposure [22, 23]. Interestingly, in our cohort patients who developed an *early-onset* AVN were mainly SLE and received a significantly lower cumulative dose of GCs, suggesting that some patients might have a high risk to develop AVN even with less GC exposure. Indeed, AVN has been more frequently reported in SLE compared to other conditions chronically treated with steroids [1]. Moreover, among patients with the same rheumatologic background and receiving a comparable dose of steroids, only a subset develops AVN, suggesting the presence of individual differences in steroid sensitivity [11].

Despite playing the major role, GCs probably are not the only factor involved in AVN pathogenesis, configuring a *multi-hit hypothesis* [24], in which different contributors may concur to the development of AVN together with GC exposure. In literature a possible synergic effect between GCs and immunosuppressive therapy has been reported^715^. In our population, a significantly greater number of patients with *early-onset* AVN was receiving GCs and at least one other DMARD at the time of AVN diagnosis. Whether this reflects a more severe rheumatologic background, requiring high-dose GCs and steroid-spearing agents, or a specific combined effect of GCs and DMARDs still needs to be elucidated. Although an increased risk for AVN has been reported in SLE patients with antiphospholipid antibodies and RNP positivity [25] and in juvenile dermatomyositis with anti-MDA5 antibodies [26], we did not find any association between specific autoimmunity in *early onset* AVN. However, larger sample studies with a control group are needed to further evaluate this aspect.

Koo et al. [11] found that the first year after the beginning of a steroidal long-term treatment are at greater risk for the development of AVN. Similarly, the study by Nakamura et al. [27] reported a rare incidence of AVN in long-term follow up of SLE patients, always associated to a flare of the disease and a subsequent increase in steroidal treatment [28]. In our cohort, patients with *early-onset* AVN were more frequently SLE and presented a high DA both at disease onset and during its course. Interestingly, only one patient presented with severe DA at the time of AVN diagnosis, while most of them had high DA during the course of the rheumatic disease, stressing the importance of a careful follow-up in all patients whose underlying condition required aggressive steroidal treatment, even once in remission.

The natural history of AVN may lead to pathological fracture of the subchondral bone with necrosis and to severe osteoarthritis in young adults due to progressive deformation of articular joints [9, 29]. Optimal treatment of AVN is controversial. A conservative strategy in case of small asymptomatic lesions can often be adequate. However, when AVN is symptomatic or affecting a sizable portion of articular joint, a surgical treatment (core decompression or free vascularized fibular grafting) could be required. Prosthetic treatment is not an ideal treatment in children and adolescents due to its possible wear and the potential risk of infection also related to long-term treatment with GCs; however, when major lesions involve a weight-bearing area a bone collapse is a common complication which may require total joint replacement. In our cohort, half of the population did not receive any treatment, with a resolution of clinical symptoms anyway, despite persistence of imaging abnormalities in almost all of them.

Our study acknowledges some limitations. Its retrospective and multicenter nature might have led to a bias in collection. Furthermore, the small sample size and the lack of a control group strongly limit our conclusion on the role of different contributors in AVN development. Nonetheless, given the extreme rarity of this condition, our cohort reflects the real-life experience of the Italian pediatric rheumatology community.

## Conclusions

AVN is a rare but severe complication of pediatric rheumatic diseases, that need to be carefully and actively monitored, to prevent long-term damage. Prospective large sample studies are required to better understand the role of GC exposure and its complex interplay with other potential contributing factors of AVN development.

## Data Availability

The dataset generated and analysed during the current study is available from the corresponding author upon reasonable request.

## Abbreviations

AVN: Avascular necrosis
SLE: Systemic lupus erythematosus
GCs: Glucocorticoids
IACI: Intra-articular steroid injection
MRI: Magnetic resonance imaging
DA: Disease activity
ANA: Antinuclear antibodies
dsDNA: Anti-double strained DNA
ENA: Antibodies, extractable nuclear antigen antibodies
ANCA: Antineutrophil cytoplasmic antibodies
IQR: Interquartile range
sJIA: Systemic juvenile idiopathic arthritis
APS: Anti-phospholipid syndrome
PAN: Polyarteritis nodosa
SAPHO: Synovitis acne, pustulosis hyperostosis, osteosis syndrome
UCTD: Undifferentiated connective tissue disease
MAS: Macrophage activation syndrome
DMARD: Disease-modifying antirheumatic drug
DMARD: Disease-modifying antirheumatic drug
